# Mechanical loading of bone-anchored implants during functional performance tests in service members with transfemoral limb loss

**DOI:** 10.3389/fresc.2024.1336115

**Published:** 2024-03-15

**Authors:** Jonathan R. Gladish, Christopher L. Dearth, Mark D. Beachler, Benjamin K. Potter, Jonathan A. Forsberg, Brad D. Hendershot

**Affiliations:** ^1^ Research & Surveillance Section, Extremity Trauma and Amputation Center of Excellence, Defense Health Agency, Falls Church, VA, United States; ^2^ Department of Rehabilitation, Walter Reed National Military Medical Center, Bethesda, MD, United States; ^3^ Department of Surgery, Uniformed Services University of the Health Sciences, Bethesda, MD, United States; ^4^ Department of Orthopaedics, Walter Reed National Military Medical Center, Bethesda, MD, United States; ^5^ Orthopaedic Service, Department of Surgery, Memorial Sloan Kettering Cancer Center, New York, NY, United States; ^6^ Department of Physical Medicine & Rehabilitation, Uniformed Services University of the Health Sciences, Bethesda, MD, United States

**Keywords:** amputation, biomechanics, kinetics, military, osseointegration, periprosthetic fracture

## Abstract

**Introduction:**

For individuals with limb loss, bone-anchored implants create a direct structural and functional connection to a terminal prosthesis. Here, we characterized the mechanical loads distal to the abutment during several functional performance tests in Service members with transfemoral (TF) limb loss, to expand on prior work evaluating more steady-state ambulation on level ground or slopes/stairs.

**Methods:**

Two males with unilateral TF limb loss and two males with bilateral TF limb loss participated after two-stage osseointegration (24 and 12 months, respectively). Tri-directional forces and moments were wirelessly recorded through a sensor, fit distal to the abutment, during six functional tests: Timed Up and Go (TUG), Four Square Step Test (FSST), Six Minute Walk Test (6MWT), Edgren Side-Step Test (SST), T-Test (TTEST), and Illinois Agility Test (IAT). Additionally, participants performed a straight-line gait evaluation on a 15 m level walkway at a self-selected speed (0.93–1.24 m/s). Peak values for each component of force and moment were extracted from all six functional tests; percent differences compared each peak with respect to the corresponding mean peak in straight-line walking.

**Results:**

Peak mechanical loads were largest during non-steady state components of the functional tests (e.g., side-stepping during SST or TTEST, standing up from the ground during IAT). Relative to walking, peak forces during functional tests were larger by up to 143% (anterior-posterior), 181% (medial-lateral), and 110% (axial); peak moments were larger by up to 108% (flexion-extension), 50% (ab/adduction), and 211% (internal/external rotation).

**Conclusions:**

A more comprehensive understanding of the mechanical loads applied to bone-anchored implants during a variety of activities is critical to maximize implant survivability and long-term outcomes, particularly for Service members who are generally young at time of injury and return to active lifestyles.

## 1 Introduction

Despite substantial technological advancements in prosthetic components, suboptimal human-device interaction can diminish functional performance and overall clinical outcomes for persons with limb loss. Specifically, residual limb tissues are not evolutionarily designed for weight bearing and thus the mechanical environment within a conventional prosthetic socket often results in poor skin health (1, 2). Poor residual skin health can limit prosthesis use, thereby reducing mobility and independence. Osseointegration, by direct skeletal attachment, mitigates many common drawbacks of conventional prosthetic sockets (e.g., inadequate fit/suspension, heat, moisture)—while also enhancing sensory feedback, movement quality, and prosthesis embodiment—for many resulting in greater prosthesis use, mobility, and quality of life (3).

Service members with limb loss are generally young at time of injury and often return to active lifestyles. To mitigate risk for periprosthetic fracture and component damage/failure (4), it is critical to understand the mechanical loads applied to the bone-anchored implant during a variety of activities. Compared to traditional biomechanical evaluations, wireless sensors incorporated into the endoskeletal prosthesis capture more direct measurement of mechanical loading at the implant-femoral interface, and can facilitate such evaluations in non-laboratory settings. Such an approach has been used to characterize forces and moments at the bone-anchored implant among individuals lower limb loss during steady-state walking (in a straight line and circles), as well as ramp/stair ascent and descent (5–7). The purpose of this study was to further characterize mechanical loading of the bone-anchored implant among Service members with unilateral and bilateral transfemoral (TF) limb loss, specifically during several functional performance tests that would be difficult to measure with traditional biomechanical methods (i.e., instrumented walkways) and are otherwise lacking in the current literature [e.g., (8)]. It was expected that mechanical loads measured during functional tasks would be larger than steady-state ambulation (i.e., walking in a straight line). Ultimately, such an effort will contribute to a more complete understanding of implant survivability in highly active populations with TF limb loss.

## 2 Methods

### 2.1 Participants

Four males with traumatic TF limb loss (Table 1), two unilateral (“UTF1” and “UTF2”) and two bilateral (“BTF1” and “BTF2”), participated after osseointegration (OPRA™ implant system; Integrum, Sweden). All participants wore microprocessor knee(s) with a dynamic response foot-ankle device(s), and were able to independently ambulate without the use of assistive devices (e.g., cane, crutches, walker). All participants consented to procedures approved by the local Institutional Review Board.

**Table 1:** Demographics for each participant with unilateral (UTF) and bilateral (BTF) transfemoral limb loss, at time of evaluation after osseointegration (OI).

		Age (yr)	Body mass (kg)	Stature (cm)	Time since amp (mo)	Time since OI (mo)
Unilateral	UTF1	50	86.5	174.5	114	24
	UTF2	42	106.0	176.0	204	24
Bilateral	BTF1	30	73.3	180.5	98	12
	BTF2	38	106.5	185.0	112	12

All participants wore microprocessor knees (X3®; Ottobock) and dynamic response ankle-foot prostheses (UTF1, Kinterra®, Proteor; UTF2, Pro-Flex® LP Torsion, Ossur; BTF1, Variflex XC®, Ossur; BTF2, Soleus Tactical®, College Park Industries).

### 2.2 Procedures

Approximately one hour prior to data collection, a certified prosthetist fit a wireless 6DOF load sensor (iPecs™; RTC Electronics Inc., Dexter, MI, USA) just distal to the failsafe mechanism (Axor II™; Integrum, Sweden). Note, both participants with bilateral TF limb loss could only accommodate a sensor on the right side due to limited clearance between the left prosthetic knee and failsafe (i.e., could not preserve limb length and alignment). Prior to data collection, the load sensor was zeroed with the prosthesis unloaded.

Tri-directional forces and moments were wirelessly recorded (850 Hz) during six functional performance tests: (1) Timed Up and Go (TUG), (2) Four Square Step Test (FSST), (3) Six Minute Walk Test (6MWT), (4) Edgren Side-Step Test (SST), (5) *T*-Test (TTEST), and (6) Illinois Agility Test (IAT). Additionally, the same forces and moments were recorded while participants completed an overground gait assessment along a 15 m walkway, at a self-selected speed. Self-selected walking speeds were 1.18 m/s (UTF1), 1.24 m/s (UTF2), 1.09 m/s (BTF1), and 0.93 m/s (BTF2).

### 2.3 Analyses

Raw forces and moments were output using the provided calibration matrix and analyzed with custom scripts in MATLAB (MathWorks, Natick, MA, USA). Raw forces and moments were normalized to body weight (BW and BW-m). The sensor was oriented such that forces and moments were resolved in anatomical coordinates of the residual limb: anterior-posterior, medial-lateral, and axial; corresponding moments in flexion-extension, ad/abduction, and internal/external rotation. Peak values within each direction/component of force and moment were identified and extracted from each functional test; for the gait evaluation, means of these peaks were computed across all steps (∼15 steps per participant). To provide a relative measure of the mechanical loads imposed during the functional tests, percent differences were computed for all peak forces and moments with respect to the corresponding mean peaks from the gait evaluation.

## 3 Results

Peak medial-lateral forces were largest in the SST, anterior-posterior forces in the IAT and T-Test, and axial forces in the SST (Table 2). Flexion-extension moments were largest in the 6MWT, ab/adduction moments in the SST, and internal/external rotation moments in the IAT (Table 3).

**Table 2:** Peak forces by functional performance test for each participant with unilateral (UTF) and bilateral (BTF) transfemoral limb loss. Forces are normalized to body weight (BW).

	Medial-lateral				Anterior-posterior				Axial			
	UTF1	UTF2	BTF1	BTF2	UTF1	UTF2	BTF1	BTF2	UTF1	UTF2	BTF1	BTF2
TUG	0.183	0.100	0.118	0.142	0.272	0.240	0.267	0.252	1.059	1.460	1.741	1.165
4SST	0.168	0.098	0.110	0.145	0.191	0.213	0.200	0.170*	1.085	1.388	1.835	1.510
6MWT	0.182	0.116	0.211	0.165	0.291	0.215	0.350	0.236	1.118	1.383	2.102	1.285
SST	0.151	0.166	0.302	0.153	0.164	0.196	0.171	0.129*	1.603	2.165	2.264	1.482
TTEST	0.196	0.148	0.232	0.153	0.308	0.276	0.279	0.220	1.326	1.971	1.978	1.563
IAT	0.193	0.121	0.148	0.188	0.419	0.233	0.546	0.255	1.280	1.688	1.888	1.250
SSW	0.163	0.090	0.108	0.134	0.239	0.193	0.225	0.160	0.867	1.094	1.077	1.042

TUG, timed up and go; FSST, four square step test; 6MWT, six minute walk test*;* SST, edgren side-step test; TTEST, T-test*;* IAT, illinois agility test; SSW, self-selected walk.

Asterisks (*) indicate two occurrences where the peak force was posterior vs. anterior for all other tests.

**Table 3:** Peak moments by functional performance test for each participant with unilateral (UTF) and bilateral (BTF) transfemoral limb loss. Moments are normalized to body weight (BWm).

	Flexion/extension				Ab/adduction				Internal/external rotation			
	UTF1	UTF2	BTF1	BTF2	UTF1	UTF2	BTF1	BTF2	UTF1	UTF2	BTF1	BTF2
TUG	0.050	0.057	0.093	0.056	0.046	0.066	0.057	0.040	0.014	0.021	0.027	0.016
4SST	0.057	0.053	0.090	0.050	0.058	0.070	0.069	0.041	0.011	0.014	0.016	0.014
6MWT	0.058	0.053	0.111	0.059	0.058	0.072	0.088	0.052	0.012	0.016	0.029	0.012
SST	0.039	0.055	0.082	0.043	0.064	0.098	0.086	0.049	0.008	0.011	0.017	0.008
TTEST	0.064	0.071	0.104	0.059	0.057	0.082	0.074	0.048	0.012	0.019	0.016	0.010
IAT	0.053	0.066	0.097	0.064	0.053	0.080	0.073	0.042	0.015	0.024	0.034	0.019
SSW	0.031	0.040	0.054	0.036	0.051	0.056	0.059	0.038	0.007	0.013	0.011	0.007

TUG, timed up and go; FSST, four square step test; 6MWT, six minute walk test; SST, edgren side-step test; TTEST, T-test; IAT, illinois agility test; SSW, self-selected walk.

**Figure 1:**
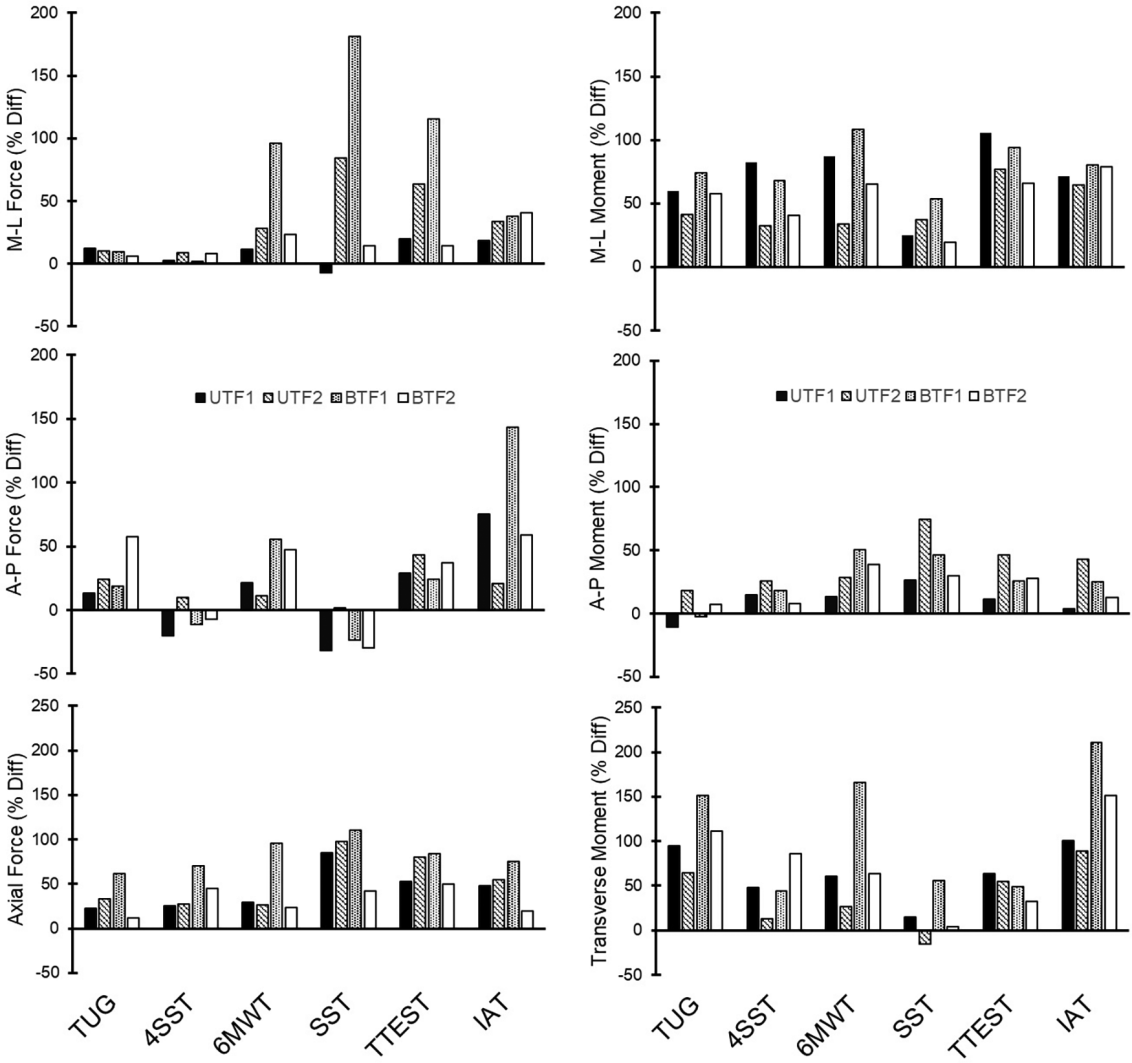
Percent differences (% Diff) in peak forces and moments, by functional performance test relative to corresponding peaks from the gait evaluation, for each participant with unilateral (UTF) and bilateral (BTF) transfemoral limb loss. TUG, timed up and go; FSST, four square step test; 6MWT, six minute walk test; SST, edgren side-step test; TTEST, T-test; IAT, illinois agility test.

Compared to straight-line walking, peak forces during functional tests were larger by up to 143% (anterior-posterior), 181% (medial-lateral), and 110% (axial; Figure 1); peak moments were larger by up to 108% (flexion-extension), 50% (ab/adduction), and 211% (internal/external rotation; Figure 1).

Persons with UTF vs. BTF generally performed better on most functional tests (Table 4).

**Table 4:** Outcomes by functional performance test for each participant with unilateral (UTF) and bilateral (BTF) transfemoral limb loss.

		TUG (s)	4SST (s)	6MWT (m)	SST (pts)	TTEST (s)	IAT (s)
Unilateral	UTF1	8.8	9.6	446.0	8	53.0	67.4
	UTF2	7.2	8.3	445.1	10	35.8	46.3
Bilateral	BTF1	9.2	12.7	496.3	9	43.7	58.0
	BTF2	14.8	17.0	347.5	7	60.2	74.7

TUG, timed up and go; FSST, four square step test; 6MWT, six minute walk test; SST, edgren side-step test; TTEST, T-test*;* IAT, illinois agility test.

## 4 Discussion

This study characterized peak mechanical loads during several functional performance tests, after osseointegration, in Service members with unilateral and bilateral TF limb loss. These peak loads tended to be largest during transient components of the functional performance tests, with peak forces and moments respectively up to 181% and 211% larger than during straight-line walking. In the sagittal plane, peak anterior-posterior forces tended to occur during initiation (e.g., standing up from the ground; IAT) or when weaving between cones (IAT); similarly, peak flexion-extension moments occurred during initiation or change of directions within the IAT and TTEST. In the frontal plane, peak medial-lateral forces tended to occur during side stepping (SST); peak ab/adduction moments also occurred during side stepping or directional changes at the far ends of the SST course. In the transverse plane, peak axial forces tended to occur during sidestepping or directional changes in the TTEST or SST; peak internal/external rotation moments occurred when turning (TUG) or weaving (IAT).

Comparing straight-line walking, peak forces and moments measured in the current study are generally comparable to prior work in persons with both transtibial and transfemoral limb loss (5–7). While smaller loads during more repetitive activities like walking (level, slopes, stairs) can accumulate over time, and thus play a role in the fatigue life of system components [e.g., perhaps necessitating prophylactic exchange/replacement; (9)], larger peak values during more transient activities remain important for minimizing unexpected breakaway or risk for unsafe load transmission. Here, despite several occurrences of non-body weight normalized peak flexion-extension moments (67–80 Nm) and internal/external rotation moments (13–25 Nm) exceeding the respective fail-safe release threshold in flexion/bending (70 ± 5 Nm) and axial twist (15 ± 2 Nm), none of these resulted in an actual release during testing. As suggested previously (7), relatively large between-subject variability supports the notion of a personalized approach to the design, prescription, and evaluation of components to adequately protect the implant and/or bone. Insufficient spacing between abutment/failsafe and prosthetic knee to accommodate the load sensor (height = 46 mm)—without affecting limb length/alignment—ultimately excluded a large majority of individuals with TF limb loss who have received osseointegration at our institution. Future work should aim to continue load characterization across a variety of activities in larger and more diverse samples (e.g., transtibial or transfemoral with other implant systems).

Of note, the four participants in the current sample were generally high-functioning per the scoring criteria of each functional performance test. For example, TUG scores here ranged from 7.2–8.8 s for UTF and 9.2–14.8 s for BTF [K3 = 12.8 ± 0.5 s and K4 = 9.5 ± 0.8 s; (10)]. 4SST scores here ranged from 8.3–9.6 s for UTF [10.4 ± 5.3 s; (11)] and 12.7–17.0 s for BTF [22.0 ± 10.2 s; (12)]. 6MWT here ranged from 348 to 496 m [K3 = 299 ± 102 m and K4 = 419 ± 86 m; (13)].

In summary, the current study which characterized mechanical loading of the bone-anchored implant during functional performance tests extends traditional biomechanical assessments (i.e., measuring ground reaction forces with force platforms, often during steady-state ambulation), and is a first step toward establishing benchmarks of peak loading during such activities. Wireless sensor approaches could enable broader surveillance of mechanical loads in the home and community, further improving ecological validity. In a larger sample, future work should also consider evaluating relationships of these mechanical loads with limb characteristics [e.g., bone quality, residual limb length; (14)], time since amputation and/or osseointegration, and prosthetic components (6, 15). Broader understanding of the mechanical loads applied to the abutment following osseointegration is critical to maximize implant survivability and long-term outcomes, particularly for Service members who are generally young at time of injury and return to active lifestyles.

## Data Availability

The original contributions presented in the study are included in the article/Supplementary Material, further inquiries can be directed to the corresponding author.

## References

[1] Koc E Tunca M Akar A Erbil AH Demiralp B Arca E. Skin problems in amputees: a descriptive study. Int J Dermatol. (2008) 47(5):463–6. 10.1111/j.1365-4632.2008.03604.x 18412862

[2] Meulenbelt HE Dijkstra PU Jonkman MF Geertzen JH. Skin problems in lower limb amputees: a systematic review. Disabil Rehabil. (2006) 28(10):603–8. 10.1080/09638280500277032 16690571

[3] Hebert JS Rehani M Stiegelmar R. Osseointegration for lower-limb amputation: a systematic review of clinical outcomes. JBJS Rev. (2017) 5(10):e10. 10.2106/JBJS.RVW.17.00037 29087966

[4] Zaid MB Wustrack RL Garibaldi M Geiger E Andaya V O’Donnell RJ. editors. Prospective study of percutaneous bone-anchored implants in transfemoral amputees: Brain-machine platform technology for external prosthetic control and feedback. 2019 9th International IEEE/EMBS Conference on Neural Engineering (NER). IEEE (2019).

[5] Frossard L Laux S Geada M Heym PP Lechler K. Load applied on osseointegrated implant by transfemoral bone-anchored prostheses fitted with state-of-the-art prosthetic components. Clin Biomech. (2021) 89:105457. 10.1016/j.clinbiomech.2021.105457 34454327

[6] Frossard L Leech B Pitkin M. Loading applied on osseointegrated implant by transtibial bone-anchored prostheses during daily activities: preliminary characterization of prosthetic feet. J Prosthet Orthot. (2020) 32(4):258. 10.1097/JPO.0000000000000280 33013144 PMC7526518

[7] Lee WC Frossard LA Hagberg K Haggstrom E Brånemark R Evans JH Kinetics of transfemoral amputees with osseointegrated fixation performing common activities of daily living. Clin Biomech. (2007) 22(6):665–73. 10.1016/j.clinbiomech.2007.02.005 17400346

[8] Niswander W Wang W Baumann AP. Characterizing loads at transfemoral osseointegrated implants. Med Eng Phys. (2020) 84:103–14. 10.1016/j.medengphy.2020.08.005 32977907

[9] Potter BK. CORR Insights®: what are the risk factors for mechanical failure and loosening of a transfemoral osseointegrated implant system in patients with a lower-limb amputation? Clin Orthop Relat Res. (2022) 480(4):732–4. 10.1097/CORR.0000000000002112 35020624 PMC8923605

[10] Sions JM Beisheim EH Manal TJ Smith SC Horne JR Sarlo FB. Differences in physical performance measures among patients with unilateral lower-limb amputations classified as functional level K3 versus K4. Arch Phys Med Rehabil. (2018) 99(7):1333–41. 10.1016/j.apmr.2017.12.033 29410114 PMC6019138

[11] Sawers A Kim J Balkman G Hafner BJ. Interrater and test-retest reliability of performance-based clinical tests administered to established users of lower limb prostheses. Phys Ther. (2020) 100(7):1206–16. 10.1093/ptj/pzaa063 32280970

[12] Carroll MK Carroll K Rheinstein J Highsmith MJ. Functional differences of bilateral transfemoral amputees using full-length and stubby-length prostheses. Technol Innov. (2018) 20(1-2):75–83. 10.21300/20.1-2.2018.75 31788157 PMC6884005

[13] Gailey RS Roach KE Applegate EB Cho B Cunniffe B Licht S The amputee mobility predictor: an instrument to assess determinants of the lower-limb amputee’s ability to ambulate. Arch Phys Med Rehabil. (2002) 83(5):613–27. 10.1053/apmr.2002.32309 11994800

[14] Taylor CE Zhang Y Qiu Y Henninger HB Foreman KB Bachus KN. Estimated forces and moments experienced by osseointegrated endoprostheses for lower extremity amputees. Gait Posture. (2020) 80:49–55. 10.1016/j.gaitpost.2020.05.018 32485424 PMC7417188

[15] Frossard L Haeggstroem E Hagberg K Brånemark R. Load applied on bone-anchored transfemoral prosthesis: characterization of a prosthesis-a pilot study. J Rehabil Res Dev. (2013) 50(5):619–34. 10.1682/JRRD.2012.04.0062 24013910

